# Network and solitude satisfaction as modifiers of disadvantages in the quality of life of older persons who are challenged by exclusion from social relations: a gender stratified analysis

**DOI:** 10.1007/s11482-022-10045-z

**Published:** 2022-03-21

**Authors:** George Pavlidis, Thomas Hansen, Andreas Motel-Klingebiel, Marja Aartsen

**Affiliations:** 1 Division of Ageing and Social Change, Department of Culture and Society, Linkoping University, Linkoping, Sweden; 2 Department of Mental Health and Suicide, Norwegian Institute of Public Health, Oslo, Norway; 3 Department for Ageing and Housing studies, Oslo Metropolitan University, Oslo, Norway; 4 Department for Ageing and Housing studies, Nova-Norwegian Social Research, Oslo, Norway

**Keywords:** Quality of life, Older persons, Social networks, Network satisfaction, Solitude satisfaction

## Abstract

This study examined from a gender-sensitive perspective the associations of exclusion from social relations (ESR) with the quality of life (QoL) of excluded older persons. Being satisfied with existing relations (i.e., network satisfaction) may be particularly important for the QoL of older persons with small networks, whereas the QoL of “network-less” older persons may be associated with their perception of solitude (i.e., solitude satisfaction). This study examined the moderating role of network satisfaction (NS) in the gendered associations between network size and QoL, as well as the gendered associations of solitude satisfaction (SS) with the QoL of older “network-less” persons. In addition, the comparative disadvantages in the QoL of “network-less” older persons with low-to-high SS, compared to the QoL of socially embedded persons with low-to-high NS were examined. Cross-sectional gender stratified secondary analyses of data from participants (N = 72.433) in the Survey on Health, Aging and Retirement in Europe (SHARE) did not provide convincing evidence that a higher NS is particularly important for the QoL of older persons with smaller networks. Among older “network-less” persons, lower SS was associated with lower QoL, comparatively more so among older women. Older persons embedded in a social network with low NS, as well as older “network-less” persons with low SS, have comparatively the lowest levels of QoL. It was concluded that the subjective evaluation of social relations and the subjective evaluation of solitude are associated with gendered disadvantages in the QoL of older persons challenged by ESR.

## Introduction

Exclusion from social relations (ESR) in older age is an unwanted situation in which older persons are socially and emotionally disconnected from adequate levels of intimate relationships, social networks, and social support (Aartsen et al., 2021). The central elements of ESR in older age are the quality of social relations and the unwanted experience of being excluded, indicated by shortages in one or more *objective* (e.g., network size) or *subjective* (e.g., perceived satisfaction from relationships) aspects that typifies social networks (Aartsen et al., 2021; Burholt et al., 2020; Walsh, Scharf, & Keating, 2017). Deficiencies in older persons’ social relations are associated with disadvantages in their health and QoL (Berkman & Glass, 2000; Courtin & Knapp 2017; National Academies of Sciences, Engineering, & Medicine, 2020), with the literature providing mixed evidence for the relative importance of the quantity versus the quality of social relations. For example, Litwin (2011) found that older persons’ social network size matters more than how satisfied they are with their established relations, whereas Pinquart & Sörensen (2000) argued for the greater importance of the quality versus the quantity of social relations in terms of older persons’ wellbeing. However, research on loneliness argues that it is both the quality and quantity that is important, as having fewer relationships than desired, as well as when the intimacy from established relationships is not realized, lead to negative effects on older persons’ wellbeing (De Jong Gierveld & Tesch-Römer, 2012; Perlman & Peplau, 1981). Yet, being objectively alone is not always equated with being emotionally afflicted, whereas loneliness is often observed among older persons with large social networks (Sundström et al., 2009).

Studies on ESR in older age suggest that those who prefer solitude or cherish their low standards of sociability may not feel lonely (Burger, 1995; Dykstra, 1995). Toyoshima and Sato (2019) found that among older persons who regard solitude positively, spending quiet time alone is associated with positive affect and better subjective wellbeing, independently from the quantity of social interactions. Using multilevel profile analyses, Lay et al., (2019) identified two types of everyday solitude among older persons, one characterized by negative affect (negative affect solitude), and one by calmness and near absence of negative affect (positive affect solitude). In their study, the desire for solitude, but not social network size, was associated with negative affect among older persons. Contrarily, several studies indicate that older persons in Mediterranean countries report higher levels of loneliness despite having larger social networks (on average) than older persons in other regions (Hansen & Slagsvold, 2016; Sundström et al., 2009; Tomini et al., 2016). As loneliness is the discrepancy between the desired and achieved levels of social relations, the differences in the prevalence of loneliness among older Europeans has been attributed to higher expectations for social interaction in some cultural settings (Sundström et al., 2009).

Newall and Menec (2019) argued that older persons’ wellbeing may depend on how the quantity and quality of their established relations act together, a topic that has received limited attention within the loneliness discourse. Litwin et al., (2015) study showed that network satisfaction (i.e., how satisfied a person is with established interpersonal relations within a social network) and network size are independent predictors of depressive symptoms among older persons. These findings give an insight into the relative importance for being satisfied with existing relations, but at the same time gives limited insight into whether satisfactory relationships are particularly important for older persons with smaller networks. Menec et al., (2020) argued that older persons that are challenged by ESR and feel lonely, as well as older persons with small networks who feel lonely, report larger support gaps and worse levels of wellbeing compared to those who do not feel lonely. The absence of loneliness among some older persons who are challenged by ESR may be attributed to their sympathetic stance towards solitary states, however, this assumption has received limited empirical scrutiny.

The disadvantages in the QoL of older persons who are dissatisfied with not having social ties may be pronounced (Umberson & Karas Montez, 2010), for whom a discussion on the relative importance of the quantity versus the quality of their social networks is probably pointless. There is little research evidence regarding older “network-less” persons’ QoL, as extreme ESR affects their likelihood of participating in relevant surveys, gaining visibility mainly in research for medical emergency cases (Newall & Menec, 2019). However, “network-less” older persons could be a target population for interventions combating ESR in older age, benefiting a small but important minority in Europe that has worse QoL compared to older persons who are socially embedded (Litwin & Stoeckel, 2013). Using longitudinal data, Litwin & Levinsky (2020) claimed that almost 400.000 older Europeans are chronically isolated from social networks and that almost five million have been in this state at least once within a period of four years. They also concluded that “*the move from a close-family interpersonal environment to a status of no-network is about as depressing as being chronically isolated*” (Litwin & Levinsky, 2020, p.11).

This study aimed to examine whether solitude satisfaction modifies the QoL of older men and women who are “network-less”, over and above other living situations that denote ESR in older age (e.g., widowhood, divorce, or living alone). We also examined whether the QoL of older persons with small social networks is particularly affected by how satisfied they are with their established relations. The gendered structure of social networks and the gendered disadvantages in socioeconomic status (SES) were considered relevant to the examination of ESR and QoL in older age. Supporting evidence derive from Schwartz & Litwin (2018), who found that older women in Europe are more likely than older men to report network expansion and lower family involvement over a period of four years. Based on meta-analysis, Pinquart & Sörensen (2000) found that compared to that of older men, social networks have a stronger influence on older women’s wellbeing. Older women are more likely than older men to experience widowhood as they live longer, with widowhood potentially leading to ESR in older age (Antonucci et al., 2001). Among widowed older women, good quality of existing relations has been associated with better mental health (Guma & Fernandez-Carro, 2021). Among older persons who live alone, women report lower health-related QoL than men (Ko et al., 2019), whereas social support has been associated with better QoL among older men but not among older women (Hajek et al., 2016). While lower SES is associated with lower social network size and less perceived available support, there is evidence of an association between the availability of support with health among older men but not among older women (Aartsen et al., 2017). Even so, and independent from gender, lower QoL is associated with lower occupational grade, ill health, and poor functional levels (Platts et al., 2015), as well as with divorce in older age (Ding et al., 2021).

Three research question guided our examination of the effects of ESR on the QoL of older persons. More precisely, we examined (i) whether network satisfaction moderates the association between network size and QoL among older persons with a network, (ii) whether solitude satisfaction is an independent predictor of QoL among “network-less” older persons, over and above other conditions that denote ESR in older age (e.g., widowhood, divorce, or living alone) and (iii) the potential disadvantages in terms of QoL of “network-less” older persons compared to those with a network, and the potential of solitude satisfaction and network satisfaction to modify these disadvantages.

Based on the literature presented above, it was hypothesized that (i) network satisfaction will moderate the relationship between network size and QoL among older persons with a network, so that network satisfaction will have stronger effects on the QoL of older persons with smaller networks, (ii) lower QoL among “network-less” older persons will be associated with lower solitude satisfaction, and (iii) older persons who are “network-less” and dissatisfied with their solitude, as well as older persons with small networks and low network satisfaction, will have worse QoL than older “network-less” persons who are satisfied with their solitude, as well as with those who are satisfied with their relations within a large network.

## Method

### Participants

Given the small sampling capacity for socially excluded older persons in large surveys (Newall & Menec, 2019; Litwin et al., 2020) the current cross-sectional study opted to maximize the sample size by pooling the data from non-institutionalized respondents in the SHARE study (*N* = 72,433) in two waves. The data were pooled from the fourth (67%) and sixth (33%) wave of the Survey for Health, Ageing an Retirement in Europe (SHARE), collected in 2011 and 2015 respectively in Austria, Germany, Sweden, the Netherlands, Spain, Italy, France, Denmark, Greece, Switzerland, Belgium, Israel, the Czech Republic, Poland, Luxemburg, Hungary, Portugal, Slovenia, Estonia, and Croatia (Börsch-Supan, 2019). Information about the procedures of the SHARE survey (e.g., sampling method, data collection method, and response rates), including ethical approval, can be found in Bergmann et al., (2019) and the official website of the survey (http://www.share-project.org/faqs/3-methodology.html). The participants included here were those who answered the social network module themselves (i.e., not via an informant). For those who participated in both waves, we used data from the fourth wave only, so no participant was included more than once in the sample. In this study sample (M_*age*_ = 66.20, *SD* = 9.79, *Range* = 50–105), 43.3% were male and 56.7% were female. The demographics of the sample disaggregated by gender, are presented in Tables 1 and 2.

**Table 1:** *Gender stratified frequency analyses of demographic, social and health variables for the study sample*

	*Men*	*Women*			*Men*	*Women*
Household size	%	%		Employment status	%	%
Living alone	15.3	28.1		Not applicable	0.4	0.7
2	59.2	51.0		Retired	60.8	51.6
3	14.5	12.6		(Self) Employed	30.4	25.1
4	7.3	5.4		Unemployed	3.7	2.9
> 4	3.6	2.9		Permanently sick	3.4	3.0
Income (euros)^a^				Homemaker	0.3	15
1st *quartile* (8614)	21.0	28.1		Other	1.0	1.7
2nd *quartile* (18,378)	24.2	25.6		Marital status		
3rd *quartile* (38,400)	26.2	24.1		Married, living with spouse	76.4	60.6
4^rth^ *quartile* (> 38,400)	28.6	22.2		Registered partnership	1.6	1.2
Chronic diseases				Married, not living with spouse	1.4	1.2
0	25.0	23.1		Never married	6.4	5.3
1	30.6	28.1		Divorced	7.7	10.2
2	21.1	21.1		Widowed	6.5	21.5
3	12.5	13.7		Network Size		
4	6.2	7.5		“Network-less” (0 members)	4.1	3.0
> 4	4.6	6.4		Small networks (1–2 members)	59.3	49.1
Mobility limitations				Large networks (≥ 3 members)	36.6	47.9
0	58.7	44.1		Solitude Satisfaction		
1	14.8	15.0		High (9–10)	30.1	28.7
2	9.0	11.0		Medium	26.7	24.2
3	5.8	8.5		Low	43.2	47.1
4	3.9	6.3				
> 4	7.9	15.0		Network satisfaction		
Limitations ADL				High (9–10)	63.6	67.5
0	91.0	89.1		Moderate (7–8)	31.9	28.7
1	5.2	5.9		Low (0–6)	4.5	3.8
2	1.7	2.4				
> 2	2.1	2.7		Limitations IADL		
CASP-12				0	88.4	89.1
< 35	29.2	34.1		1	6.4	5.9
≥ 35	79.8	65.9		2	2.2	2.4
				> 2	3.1	2.7

*Note.* CAPS-12 = Quality of life Index, ADL = Activities of daily living, IADL = Instrumental activities of daily living,

^a^ Household income quartiles

**Table 2:** *Gender differences (t-test) and gender stratified Pearson correlation coefficients for demographics, health, social relations, and CASP-12 scores*

	Men		Women		Gender _Differences_	CASP-12 (*r*)	
	*M*	*SD*	*M*	*SD*	*t*	Men	Women
Age	66.27	9.45	66.15	10.03	1.690	− 0.133^*^	− 0.167^*^
Household size	2.27	1.00	2.06	1.00	27.710^*^	− 0.006	0.018^*^
Household income^a^	35.87	65.26	30.26	63.09	11.690^*^	0.171^*^	0.175^*^
Years of education	11.26	4.42	10.56	4.23	21.582^*^	0.170^*^	0.219^*^
*N* of chronic diseases	1.61	1.47	1.78	1.58	-14.510^*^	− 0.280^*^	− 0.328^*^
*N* of mobility limitations	1.17	1.95	1.87	2.38	-41.924^*^	0.400^*^	0.421^*^
Limitations ADL	0.17	0.67	0.21	0.75	-7.808^*^	− 0.244^*^	− 0.256^*^
Limitations IADL	0.25	0.89	0.40	1.05	-20.226^*^	− 0.295^*^	− 0.331^*^
Social network size	2.28	1.5	2.67	1.60	-33.146^*^	0.105^*^	0.163^*^
Network satisfaction	8.80	1.46	8.93	1.38	-11.924^*^	0.172^*^	0.176^*^
Solitude satisfaction	5.29	3.98	5.31	3.87	− 0.179	0.238^*^	0.365^*^
CASP-12	37.51	6.13	36.67	6.52	17.762^*^	-	-
*Note.* CAPS-12 = Quality of life Index, ADL = Activities of daily living, IADL = Instrumental activities of daily living ^a^ in thousand euros ^*^ *p* = .000							

## Measures

***Network size.*** The size of older persons’ social network was assessed in SHARE by using a name generating inventory. Participants were asked to name up to six persons with whom they had discussed important issues within the last year, using the following probe question “*Over the last 12 months, who are the people with whom you most often discussed important things? … These people may include your family members, friends, neighbours, or other acquaintances*”. Respondents were also given the opportunity to list an additional person who is important to them for any other reason (Börsch-Supan, 2019). Older persons who scored 0 in this inventory were considered as “network-less”, according to previous research (Litwin & Levinsky, 2020). Older persons reporting one or two members in their social network were categorized as having a small social network, and those reporting three or more members in their social network were categorized as having a large social network.

***Network satisfaction.*** For those reporting at least one person in their social network, network satisfaction was assessed using a single question: “*Overall, on a scale from 0 to 10, where 0 means completely dissatisfied and 10 means completely satisfied, how satisfied are you with the [relationship that you have with the person/relationships that you have with persons] we have just talked about?*” (Börsch-Supan, 2019). As observed in previous studies using SHARE data (Litwin et al., 2015), network satisfaction was highly skewed (*M* = 8.75, *SD* = 1.70), with 43% reporting that they were completely satisfied with their relations. Therefore, a dummy variable was created to represent low (0–6), medium (7–8), and high (9–10) network satisfaction.

***Solitude satisfaction.*** For those who did not report a person in their network (i.e., “network-less”), SHARE used the probe “*You indicated that there is no one with whom you discuss important matters, and no one who is important to you for some other reason. On a scale from 0–10, where 0 means completely dissatisfied and 10 means completely satisfied, how satisfied are you with this (situation)?*” (Börsch-Supan, 2019). The solitude satisfaction scale has the same scoring principles as the network satisfaction scale, indicating low (0–6), medium (7–8), and high (9–10) satisfaction with being in a solitary state.

***Quality of life.*** The CASP-12 scale was used in SHARE to measure QoL (Börsch-Supan, 2019). The scale has twelve items rated on a four-point Likert-scale, from “never” to “often”. The items represent four dimensions of QoL, namely (i) control, defined as the ability to actively intervene in one’s environment, (ii) autonomy, defined as the ability of an individual to be free from the unwanted interference of others, (iii) self-realization, the active process of self-fulfilment, and (iv) pleasure, the reflective and active process of self-fulfilment. The total score on the CASP-12 scale ranges from 12 to 48, with higher scores indicating better QoL. Cronbach’s *α* for the CASP-12 scale in the SHARE data was reported to be *a* = 0.833 (Oliver et al., 2021).

***Demographics.*** Participants in the SHARE study were asked whether any other person was living in their household and the number of household members. Participants reporting 0 cohabitants were regarded as living in a single household. Household income was imputed based on total household income, household net worth, total household expenditure plus characteristics of the household respondent, available at the gv_imputation module of the database. Education was measures by asking about the number years spent attending full-time education. Participants were also asked to state whether they were married and cohabiting with their partner, were in a registered partnership, were married but not cohabiting with their partner, had never been married, were divorced, or were widowed. Participants were also asked about their employment status, with the possible options of being retired, employed or self-employed, unemployed, permanently sick, a homemaker, or other (Börsch-Supan, 2019).

***Health.*** The number of chronic health conditions (Range: 0–12) was an imputed variable available in the gv_health module of the SHARE study, containing a broad range of physical and mental health measures and indices. Participants were also asked about the limitations (Range: 0–6) they experience with six activities of daily living (ADL), namely dressing, walking across a room, bathing, eating, getting in or out of bed, and using the toilet. Participants were asked about the limitations (Range: 0–7) they face with seven instrumental activities of daily living (IADL), namely using a map, preparing a hot meal, shopping for groceries, making telephone calls, taking medications, doing work around the house or garden, and managing money. In addition, participants were asked whether they have any mobility limitations (Range: 0–10) in ten activities including arm function and fine motor limitations. For all variables, higher scores indicate worse health (Börsch-Supan, 2019).

## Analytical strategy

A preliminary analysis examined statistically significant gender differences in all study variables, as well as in the representation of men and women among the “network-less” with low-to-high solitude satisfaction. The moderation effect of network satisfaction in the association between network size and QoL was examined separately for men and women through gender stratified regression analysis. Following the recommendations of Frazier et al., (2004), network size and network satisfaction were entered in one step as independent predictors, and their interaction term (size*satisfaction) in a subsequent step in the regression models. To examine whether solitude satisfaction predicts independently the QoL of older “network-less” men and women, gender stratified regression analysis was conducted. In all regression models, education, income, marital status, employment status, and health indicators were added in different steps.

**Fig. 1:**
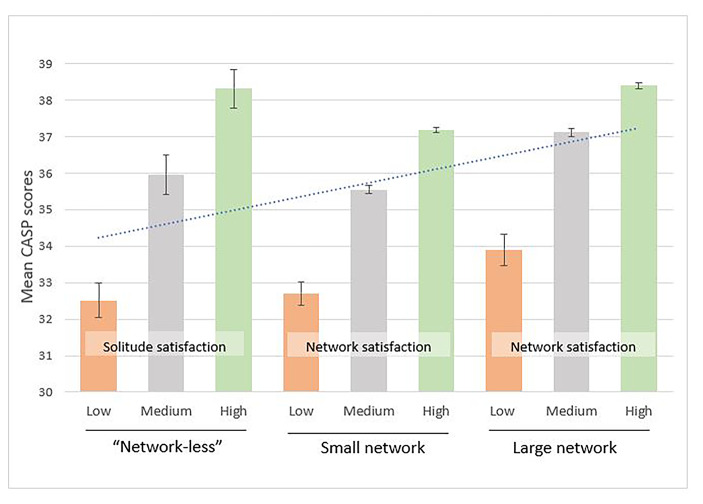
Mean scores and 95% confidence intervals in the CASP-12 scale, across nine categories formed by network size, network satisfaction, and solitude satisfaction

To examine statistically significant differences in the CASP-12 scores between older persons who are (i) “network-less”, (ii) embedded in small networks, or (iii) embedded in large networks, an Analysis of Variance (ANOVA) was conducted. Older “network-less” persons were divided in three groups representing low-to-high solitude satisfaction. Older persons with small networks were divided into three groups representing low-to-high high network satisfaction. Similarly, older persons with large networks were divided in three groups representing low-to-high high network satisfaction. In total, nine groups were formed (see Fig. 1) representing “network-less” older persons with low-to-high solitude satisfaction (three groups), older persons with small networks and low-to-high network satisfaction (three groups), and older persons with large networks and low-to-high network satisfaction (three groups). The assumption of normal distribution and homogeneity of variance was violated on various occasions in the ANOVA and post-hoc analyses. Following the recommendations of Zimmerman (1998), ANOVA was preferred over non-parametric tests. All data were analysed using SPSS v.27.

## Results

***Preliminary analyses.*** Descriptive analyses revealed that among older persons with a network a small proportion of men and women reported low network satisfaction (men = 4.5%, women = 3.8%, see Table 1), whereas a disproportionately higher percentage of older “network-less” persons reported low solitude satisfaction (men = 43.2%, women = 47.1%, see Table 1). The results of a preliminary analyses on gender differences in demographics, health, social network indicators and the CASP-12 scores, along with correlation coefficients of demographics, health, social network indicators with CASP-12 scores are reported in Table 2. Pearson chi square analysis did not reveal any significant dependency [*χ*^2^ (1, *N* = 2143) = 3.404, *p* > .050] of gender on the three solitude satisfaction categories (i.e., low, medium, and high). The mean CASP-12 scores across the three groups of network size and per category of network or solitude satisfaction, along with the 95% confidence intervals for each group, are presented in Fig. 1.

**Fig. 2:**
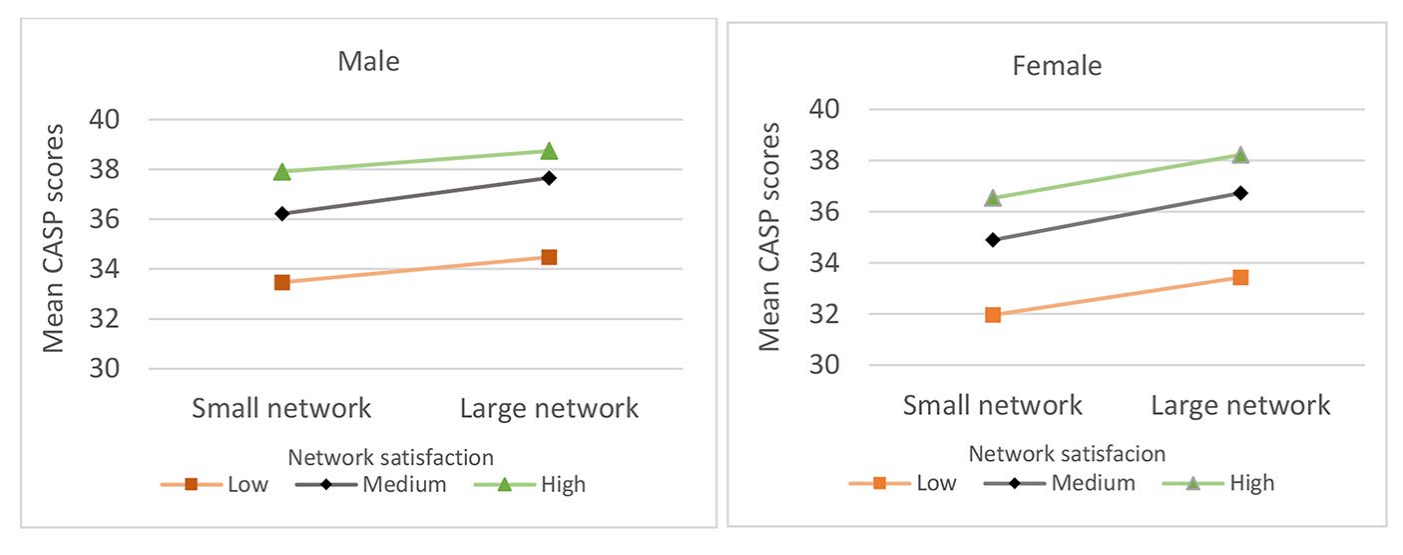
Mean scores in the CASP-12 scale, across categories of network size and network satisfaction, indicating no interaction of older persons’ network satisfaction in the associations of their network size with their CASP-12 scores

***The moderation effect of network satisfaction in the gendered associations of network size with QoL.*** The gender stratified linear regression analyses for the CASP-12 scores among older persons *with a network*, using demographics, health variables, network size, network satisfaction, as well as their interaction term (size*satisfaction) as predictors, yielded statistically significant models for both men (*A*R^2^ = 24.9%, *p* = .000) and women (*A*R^2^ = 29.9%, *p* = .000; see Table 3). For both genders, the demographic variables (R^2^_change_ = 9.4% for men, R^2^_change_ = 11.1% for women) and the health variables (R^2^_change_ = 12.8% for men, R^2^_change_ = 14.6% for women) explained most of the variance in the CASP-12 scores. Network satisfaction (*β*_men_ = 0.172, *β*_women_ = 0.165) and network size (*β*_men_ = 0.068, *β*_women_ = 0.098) emerged as independent predictors of CASP-12 scores, which together explained an additional variance of 2.7% for men and 3.6% for women. The network size*network satisfaction interaction term also emerged as an independent predictor (*β*_men_ = 0.046, *β*_women_ = 0.032) of CASP-12 scores, marginally explaining an additional variance for both men and women (R^2^_*change*_ = 0.1%). A visual inspection of Fig. 2 and the small variance explained by the interaction term in the models suggests trivial or no moderation effects of network satisfaction in the association of network size with the CASP-12 scores for both genders.

**Table 3:** *Standardized beta coefficients of the linear regression models with demographics, health variables, network size, network satisfaction, and solitude satisfaction as predictors of CASP-12 scores among older persons with or without a network*

	Without a network			With network	
	Men	Women		Men	Women
***1st block (R*** ^***2***^_***change***_ ***)***	10.7%**	16.3%**		9.4%**	11.1%**
Age	− 0.003	0.070*		− 0.014*	0.054**
Living alone	0.084*	0.031		− 0.013	− 0.051**
Household income	0.110**	0.165**		0.114**	0.101**
Education	0.127**	0.117**		0.087**	0.092**
Marital status^a^					
Registered partner	− 0.001	0.055*		0.003	0.011**
Married. no cohabiting	0.013	0.017		− 0.009	− 0.026**
Never married	0.059	0.039		− 0.005	− 0.025**
Divorced	0.017	− 0.051		− 0.002	− 0.054**
Widowed	0.010	0.022		− 0.007	− 0.061**
Employment status^b^					
(Self) Employed	0.042	0.040		0.004	0.017**
Unemployed	− 0.091**	− 0.008		− 0.085**	− 0.051**
Permanently sick	− 0.058*	− 0.032		− 0.052**	− 0.027**
Homemaker	− 0.001	− 0.112**		− 0.021**	− 0.057**
***2nd block R*** ^***2***^_***change***_	10.0%**	15.0%**		12.8%**	14.6%**
*N* of chronic diseases	− 0.110**	− 0.105**		− 0.119**	− 0.125**
*N* of mobility limitations	− 0.203**	− 0.246**		− 0.264**	− 0.291**
*N* of limitations ADL	0.056	0.007		0.018**	0.018**
*N* of limitations in IADL	− 0.128**	− 0.114**		− 0.098**	− 0.102**
***3rd block R*** ^***2***^_***change***_	3.7%**	6.4%**		2.7%**	3.6%**
Solitude satisfaction	0.194**	0.260**			
Network satisfaction _(z−score)_				0.172**	0.165**
Network size _(z−score)_				0.068**	0.098**
***4th block R*** ^***2***^_***change***_				0.1%**	0.1%**
Network satisfaction * size _(z−score)_				0.046**	0.032**
***AR*** ^***2***^	**23.2%**	**36.6%**		**24.9%**	**29.9%**
*Note.* ADL = Activities of daily living, IADL = Instrumental activities of daily living. Network satisfaction and network size was included only in the models examining the CAPS-12 scores of older persons with a network. Solitude satisfaction was included only in the models examining the CASP-12 scores of older persons without a network. ^a^ Reference group = married living with spouse ^b^ Reference group = retired, ^*^ *p* < .050, ^**^ *p* < .010					

***Gendered associations of solitude satisfaction with the QoL of older “network-less” persons***. The gender stratified linear regression analyses for older *“network-less”* persons’ CASP-12 scores with demographics, health variables, and solitude satisfaction as predictors yielded statistically significant models for both men (*A*R^2^ = 23.2%, *p* = .000) and women (*A*R^2^ = 36.6%, *p* = .000; see Table 3). For both genders, the demographic variables (R^2^_change_ = 10.7% for men, R^2^_change_ = 16.3% for women) and the health variables (R^2^_change_ = 10% for men, R^2^_change_ = 15% for women) explained most of the variance in CASP-12 scores. Solitude satisfaction emerged as an independent predictor (*β*_men_ = 0.194, *β*_women_ = 0.260), explaining an additional variance of 3.7% and 6.4% for men and women.

**Fig. 3:**
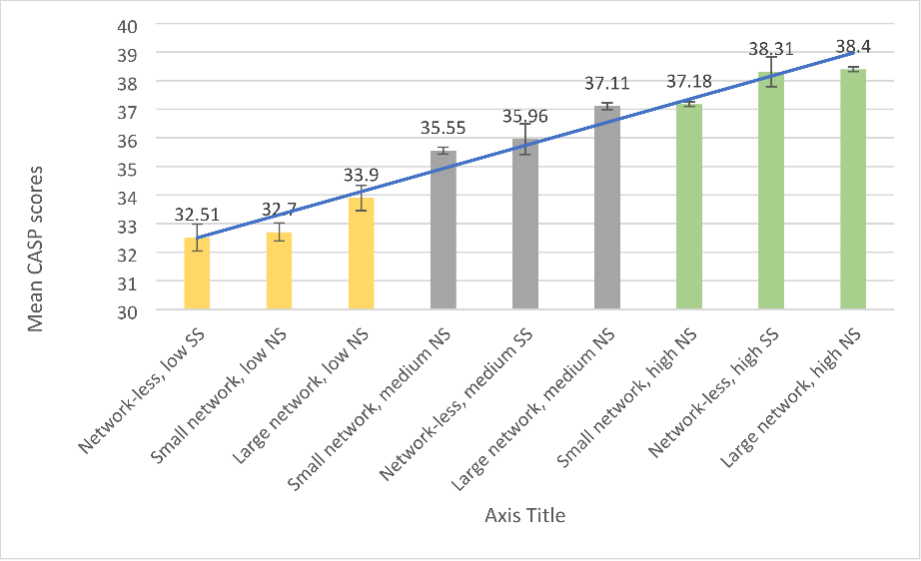
Gradient of mean scores and 95% confidence intervals in the CASP-12 scale, across groups of older persons formed by network size, network satisfaction, and solitude satisfaction *Note*: SS = Solitude satisfaction, NS = Network satisfaction

***Comparison of CASP-12 scores***. A one-way ANOVA analysis examined statistically significant differences in CASP-12 scores among nine groups of older persons, formed by distinctive constellations of network size (i.e., “network-less”, small networks, large networks) with low-to high solitude or network satisfaction, yielding a statistically significant model [*F*(8, 69,307) = 401.978, *p* = .000]. The results of the subsequent post-hoc Tukey analyses showed a solitude or network satisfaction dependent gradient where older “network-less” persons with low solitude satisfaction, as well as older persons with small or large networks and low network satisfaction had statistically significant lower CASP-12 scores compared to the rest of the groups (see Table 4; Fig. 3).

**Table 4:** *Mean differences (I-J) on the CASP-12 scores between groups of older persons that have none, small (one or two members), or large (three or more members) social networks, divided in solitude satisfaction and network satisfaction categories*

		“Network-less” (J)			Small network (J)				Large network (J)		
“Network-less” (I)		Low^a^	Medium^a^	High^a^	Low^b^	Medium^b^	High^b^		Low^b^	Medium^b^	High^b^
Low^a^		-	-3.446*	-5.800*	− 0.195	-3.040*	-4.671*		-1.386*	-4.602*	-5.889*
Medium^a^			-	-2.355*	3.251*	0.406	-1.225*		2.059*	-1.156*	-2.444*
High^a^				-	5.606*	2.760*	1.130*		4.414*	1.198*	− 0.089
Small network (I)											
Low^b^					-	-2.845*	-4.476*		-1.192*	-4.407*	-5.695*
Medium^b^						-	-1.631*		1.654*	-1.562*	-2.849*
High^b^							-		3.284*	0.069	-1.219*
Large network (I)											
Low^b^									-	-3.215*	-4.503*
Medium^b^										-	-1.288*
High^b^											-
*Note.* Negative values indicate lower scores, and positive values indicate higher scores in the CASP-12 scale for the (I) column category. ^a^ Refers to solitude satisfaction ^b^ Refers to network satisfaction ^*^ *p* = .000											

## Discussion

This study set out to examine whether satisfactory relations are particularly important for the QoL of older persons with small networks. We also examined whether the QoL of older persons who are “network-less” is associated with their evaluation for their solitary state, over and above the effects of other conditions that denote ESR in older age (e.g., widowhood, divorce, or living alone). Lastly, we compared “network-less” older persons and older persons with small networks to those who have large networks, examining whether solitude satisfaction and network satisfaction modify their comparative disadvantages in QoL.

Within the strengths and limitations of the SHARE study in terms of capturing the social milieu of older persons in Europe, we found that about 4% of older men and 3% of older women do not have a network. Among the “network-less” older persons, a significant proportion (appox. 55%) are highly or moderately satisfied with their solitude. This is consistent with previous findings arguing that withdrawal from social ties is not always experienced as an unpleasant situation, possibly reflecting an expression of personality characteristics that favour “positive” solitary states in older age (Burger, 1995; Dykstra, 1995; Lay et al., 2019; Newall & Menec, 2019; Toyoshima & Sato, 2019). There were no gender differences in the perception of solitude among “network-less” persons, suggesting that solitude is perceived negatively just as frequently among older men and women. One note of caution is that while 43% precent of older “network-less” men and 47% of older “network-less” women were dissatisfied with their solitude, only 4.5% of older men and 3.8% of older women were dissatisfied with their social network, indicating that social relations can be less challenging than being excluded from social relations in older age.

Not supporting our study’s first hypothesis, network satisfaction was equally important across groups of older persons with smaller and larger networks, suggesting greater effects of network satisfaction over network size on the QoL of older persons. Consistent with the findings of Tomini et al., (2016), the results indicate that smaller networks are associated with lower levels of QoL among older persons, with the effects being approximately 50% larger among women. Echoing previous research (Litwin et al., 2015; Pinquart & Sorensen, 2000) the findings indicate that network satisfaction modifies the QoL of older persons to a greater extent than the size of their social network. Supporting our study’s second hypothesis, the evidence indicates that among older “network-less” persons, lower solitude satisfaction predicts independently worse QoL in older age, over and above other indicators of ESR (i.e., widowhood, divorce, living alone). The findings indicate that solitude satisfaction is particularly important for the QoL of older “network-less” women, as the proportion explained on QoL was approximately to 50% increased among older women compared to older men.

Supporting the study’s third hypothesis, the findings indicate disadvantages in the QoL of “network-less” persons with low solitude satisfaction, as well as disadvantages in the QoL of older persons who are not satisfied with their existing relations. These groups had the lowest levels of QoL with a marginal difference between them, indicating that the “unwanted” nature of ESR in older age is reflected in aspects that go beyond the quantity of social relations. Accordingly, the findings of our study indicate that older “network-less” persons who regard their solitude positively have better QoL than most older persons who are moderately or not at all satisfied with their social relations. This is consistent with previous research (Menec et al., 2020) indicating that ESR in older age may have multiple facets that act together, with the perception of solitary states and the evaluation of social relations being important aspects that modify disadvantages in the QoL of excluded older persons.

The findings of our study may have potential implications for interventions that aim to tailor actions to counter the negative consequences of ESR in older age (Fakoya et al., 2020). Older persons who are dissatisfied with their solitary state are motivated to engage in more social interactions (Menec et al., 2020). Relevant interventions have predominately aimed to create opportunities to expand older persons’ existing social networks, through digital communication solutions or other structured actions (Baker et al., 2018; Poscia et al., 2018). Such activities may be an appealing option for those who are challenged by the unwanted nature of ESR in older age, however, it would probably be an unappealing option for those who feel “better off” alone. The evidence of this study indicates that the QoL of older persons who prefer their solitude may not be affected as intuitively expected, however, setting up a discrete supportive network that can react in the event of emergencies amongst them should be considered (Newell & Menec, 2019; Newall 2015).

For older persons who are embedded in a social network but are dissatisfied with their interpersonal relations, resolving relational issues may have more promising outcomes. The importance of having good-quality relations has been stressed as an indispensable part of a good life in older age (Ryff, 1989), and emerged within the COVID-19 pandemic as a pivotal condition for the wellbeing of older persons (Cavallini et al., 2021; Macdonald & Hülür, 2021). The evidence of our study indicates that assessing solely the quantity of older persons’ social network could lead to misleading conclusions and probably to less suitable interventions for older persons who may be excluded from social relations or social networks that satisfy their expectations.

It should be noted that the term “network-less” as used in this study did not refer to older adults’ seclusion from any form of human contact. The social network inventory used in SHARE makes inquiries about older persons’ confidants plus a person who is important for them for any reason. Other indicator of ESR in older age, such as widowhood, divorce, and living alone, were included in our models as separate factors that exert independent effects on the QoL of older persons. Even so, older persons’ perceptions of solitude and the evaluation of their own network remained important aspects that modify disadvantages of the wellbeing of excluded older persons. Future studies could consider older persons’ participation in wider social activities (e.g., participation in clubs, etc.) and whether these compensate for social needs created among older persons who are, and feel, less privileged in terms of intimate relations. The subjective evaluation of these activities (e.g., satisfaction from social activities) should be also considered in tandem.

As a conclusion, this study adds to the discussion of the gendered outcomes of ESR in older age, by highlighting the role of network satisfaction and solitude satisfaction for the QoL of older persons. It evidences that solitude is not always perceived unfavourably, although regarding solitude negatively seems to be associated with lower QoL in older age, especially among older women. While larger social networks may be beneficial for the QoL of older persons, evaluating positively existing relations seems to be comparatively a stronger predictor of QoL in older age. We conclude that the subjective evaluation of social relation among older persons embedded in a social network, and the evaluation of solitude satisfaction among “network-less” older persons, are important factors associated with gendered disadvantages in the QoL of older persons.
